# The Effects of Indoor High Temperature on Circadian Rhythms of Human Work Efficiency

**DOI:** 10.3390/ijerph16050759

**Published:** 2019-03-02

**Authors:** Guozhong Zheng, Ke Li, Wentao Bu, Yajing Wang

**Affiliations:** School of Energy, Power and Mechanical Engineering, North China Electric Power University, Baoding 071003, China; like@ncepu.edu.cn (K.L.); buwentao@ncepu.edu.cn (W.B.); wangyajing@ncepu.edu.cn (Y.W.)

**Keywords:** indoor high temperature, work efficiency, circadian rhythm, cosinor method, response time

## Abstract

Indoor non-air-conditioned environments widely exist in the summer high temperature weather. The work efficiency of the people who stay indoors for a long time is seriously affected by the indoor high temperature. In this paper, the changes of the circadian rhythms of work efficiency in indoor high temperature environments were studied. Ten healthy subjects (five males and five females) were selected in the experiments randomly. In each experiment day, the maximum hourly outdoor temperature was selected as 28 °C, 32 °C, 36 °C, and 38 °C, respectively, to determine the experiment conditions. In each experiment condition, subjects’ response time, accuracy rate, grip strength, work willingness, and physiological parameters were monitored for 24 consecutive hours. Meanwhile, the hourly outdoor temperatures of the experiment day were accessed from the weather report during the experiment. Then the cosinor method and statistical method were adopted. The results indicated that the response time, grip strength, and work willingness followed circadian rhythms. However, the accuracy rates of the Stroop color-word test (SCWT) and numeral inspection task (NIT) did not show an obvious circadian rhythm. The effects of high temperature on the circadian rhythms of grip strength and work willingness were mainly reflected in the decreases of the median and amplitude. The effects on the response time were mainly reflected in the decrease of the median. In addition, forehead temperature showed a significant negative correlation to response time, and it could be considered as a predictor to assess the level of work efficiency. This study gives an alternative method to replace direct measurement of the ability indices at work site and provides basic data of 24 consecutive hours, for showing changes in human work efficiency. It could be helpful to predict the low performance in advance to reduce occupational accidents.

## 1. Introduction

Biorhythm is the body’s adaptability to the expected changes in environmental conditions [1]. The ability indices, such as work efficiency and response time show a characteristic of circadian rhythms [2,3,4]. Body’s physiological and biochemical processes (such as heart rate, blood pressure, and body temperature) generally have temperature dependence, and they change with environment temperature [5,6,7]. Although the temperature in a certain range does not affect the circadian rhythms because of the temperature compensatory, the effects of the temperature on the regulation of circadian rhythms depend on the severity of environmental challenges [8]. 

In recent years, the summer high temperature weather appears in high frequency and high intensity. Some places (such as school classrooms and student dormitories) cannot afford the installation and operation of air conditioners, and some other places (such as security pavilion and other remote places) are not suitable to install air conditioners, therefore people have to endure the indoor high temperature for a long time. Their behavioral ability and physical health are both adversely affected by high temperature. Their body thermal balance is affected, then the body temperature gradually increases, and as a result, the nerve reflex latency will then increase. As a result, the action sensitivity and coordination will decrease [9,10]. The prolongations of response time and action time are important factors that influence health safety and cause accidents [2,11,12]. As a basic characteristic of the human body, the study of circadian rhythm is of great significance in daily life, health care, diseases treatment, and work efficiency improvement. Moreover, the use of circadian rhythms is conducive to predict the low performance in advance, and it can be used to minimize the accidents caused by human factors [13].

Some scholars have studied the effects of high temperature on work efficiency. Tian et al. [14] studied the work efficiency in a battle plane cabin under high temperature environments. They simulated two temperature conditions (40 °C and 45 °C) in a chamber, and 10 male subjects without heat acclimatization were selected. Subjects were asked to conduct tests in terms of perform grip, perception, finger flexibility, response time, and intelligence every 20 min, then the test data were compared with the composite heat stress index (CIHS). The results showed that CIHS exceeded the heat stress safety line at 45 min and 20 min, the grip value decreased by 12% and 3.2%, and the minimum perceived force increased by 2.89 and 4.36 times, respectively. The finger flexibility was less affected, the response speed accelerated in the initial stage and then decreased with time, the mental test time shortened, and the error rate increased. Yan et al. [15] studied the effects of passive high temperature on the human attention system using the Attention Network Test (ANT). Sixteen right-handed healthy volunteers were enrolled in the high-temperature group and the control group. In the high-temperature cabin, the temperature was 50 ± 1 °C and the relative humidity was 40 ± 1%. While in the control group, the temperature was 20 ± 1 °C and the relative humidity was 40 ± 1%. The ANT test was conducted, and the response time, core body temperature, and body weight of the subjects were also measured. Variance analysis showed that the overall response times and accuracy rates between these two groups showed no significant difference. And high temperature had obvious effects on the executive control function, while it had no significant effect on alertness and orientation. Cui et al. [16] studied the influences of indoor temperature on human work efficiency. They simulated steady-state environments at 22 °C, 24 °C, 26 °C, 29 °C, and 32 °C in an environmental chamber. 36 subjects (18 males and 18 females) were divided into group A and group B, and group A was exposed to above 5 temperature conditions, while Group B was only exposed to the temperature condition of 26 °C, then the work efficiency was measured by the number of correct letters in a memory typing task. The results indicated that the relationships between the temperature and the work efficiency showed the trend of a U-shaped curve. And the best working temperature was 25.8 °C. Hancock et al. [17] concluded that the significant decline in mental exercise and perceptual tasks was associated with heat stress. In addition, the task type, exposure duration, and stress intensity were the key variable factors affecting work efficiency in thermal environments. Zhang et al. [18] studied the effects of higher temperature in summer on office workers’ cognitive load and thermal comfort in Australia. Twenty-six office workers were tested in a climate chamber at 22 °C and 25 °C, respectively. And the tests included the Cambridge Brain Science (CBS) Cognitive Performance, NASA Task Load Index (NASA-TLX), and questionnaires about thermal comfort and air quality, meanwhile electroencephalogram (EEG) and heart rate (HR) were also measured. The results indicated that the CBS test scores were not significantly affected by high temperature, and the cognitive load significantly reduced at 25 °C, however, EEG, HR, and thermal comfort at different temperatures all showed no significant difference. 

Some scholars have focused on the relationships between circadian rhythms and human cognition. Lu [19] studied the impact of blood pressure rhythm on cognition. Ninety patients with vascular cognitive impairment were selected as subjects, and their ambulatory blood pressure was monitored for 24 h. In addition, the Montreal cognitive scale assessment was performed. The results showed that the changes of blood pressure rhythm had a significant effect on the patients’ overall cognitive function, and the vascular cognitive function was significantly affected by the diurnal mean systolic pressure, while the diurnal mean diastolic pressure did not significantly impair the cognitive function. Xu et al. [20] studied the relationships between the circadian rhythm of blood pressure and the cognitive function of elderly hypertensive patients. A total of 292 elderly hypertensive patients were selected as the experimental group and 100 healthy individuals were selected as the control group. All subjects’ cognitive function was assessed by the Mini-Mental State Examination (MMSE). The results indicated that the MMSE score of the hypertension group was lower than that of the control group. And the decrease or disappearance of circadian rhythm of blood pressure was associated with the impairment of cognitive function of elderly hypertensive patients. Morris et al. [13] argued that there was a parallel relationship between circadian rhythms and human behavioral awareness, and chronobiological changes during the simulated nightshift can be used to predict the performance awareness. Thun et al. [21] reviewed the effects of circadian rhythm and sleep on body athletic performance. The results concluded that the athletic performance seemed to be the best when the core body temperature typically was at its peak at the late afternoon. Sports involving technical skills seem to have an acrophase somewhat earlier (i.e., in the afternoon) than that of muscle strength and anaerobic performance. For technical skills related to precision control, their ability peak appeared earlier in the day.

From the above literature, previous studies mainly focused on the effects of high temperature on human work efficiency and the relationships between circadian rhythms and brain cognition. However, researches on the effects of high temperature on the circadian rhythms of human work efficiency are not common. In addition, it is difficult and fussy to measure the ability indices at work sites. Lacking studies were conducted in exploring an alternative method to replace direct measurement of ability indices. In this paper, the effects of indoor high temperature on circadian rhythms of human performance were studied. Response time, subjective feelings and grip strength were adopted to evaluate the physical performance. Then the inherent relationships between the physiological and ability parameters were also analyzed to find an easier method to replace direct measurement of ability indices. 

The aims of this paper are reflected in three aspects. The first is to explore an alternative method to replace the direct measurement of ability indices at work site. The second is to develop a comprehensive method to evaluate the human performance in indoor hot environments. The third is to predict the low performance in advance for minimizing accidents caused by human factors.

## 2. Materials and Methods

### 2.1. Experiment

#### 2.1.1. Subjects and Ethics Approval

Ten college students were selected (including 5 males and 5 females) as the subjects. The anthropometric information of the subjects is shown in Table 1. When selecting the subjects, the subjects were required to be in good mental and physical health. During the experiment, all subjects were dressed in a short sleeve T-shirt, thin pants, underwear, socks, and slippers, and the thermal resistance of clothing was 0.5 clo. In addition, caffeine and alcohol were prohibited during the experiment.

All participants provided written informed consent. The study was approved by the Chinese Ethics Committee of Registering Clinical Trials (No. ChiECRCT-20170108).

#### 2.1.2. Experiment Condition

The experiment was conducted in the top floor of a non-air-conditioned house in Baoding during July to August 2017, and natural ventilation was kept during the experiment. As there is a positive correlation between temperature and heat-related diseases [22], the daily temperature condition was determined based on the maximum hourly outdoor temperature of the experiment day. And the temperature conditions were set as 28 °C, 32 °C, 36 °C, and 38 °C (the maximum hourly outdoor temperature ± 1.0 °C), respectively. The minimum interval between each temperature condition was 4 days and each temperature condition were an experiment day. As this paper only studied the effect of the temperature on the circadian rhythms of human work efficiency, the effect of the relative humidity was not considered. All these four temperature conditions were selected on sunny days, as the variation in the hourly temperature curves were relatively similar [23,24]. To determine the experiment day of a certain temperature, the weather forecast were consulted before an experiment day. When the forecasted maximum temperature met the requirement of the temperature condition, the experiment at this temperature condition was conducted in the next day. In addition, the hourly outdoor temperatures of the experiment day were accessed from the website of China Weather [25] during the experiment day. When the maximum temperature met the requirement of the certain temperature condition, the experiment at this temperature condition was completed. Otherwise, the experiment at this temperament condition was redone [23,24].

#### 2.1.3. Work Efficiency Test

In this study, response time, accuracy rate, grip strength, and work willingness were adopted to evaluate work efficiency. The response time and accuracy rate were measured by the Stroop color-word test (SCWT) and numeral inspection task (NIT). The SCWT, originally developed by Stroop [26] in 1935, is mostly used to measure the individual’s ability to shift cognitive set and the cognitive inhibition [27]. Grip strength was used to evaluate the physical ability. In addition, self-evaluation questionnaire of the willingness to work and the body physiological parameters (systolic pressure, diastolic pressure, heart rate, rectal temperature, eardrum temperature, and forehead temperature) were also measured.

**Figure 1:**
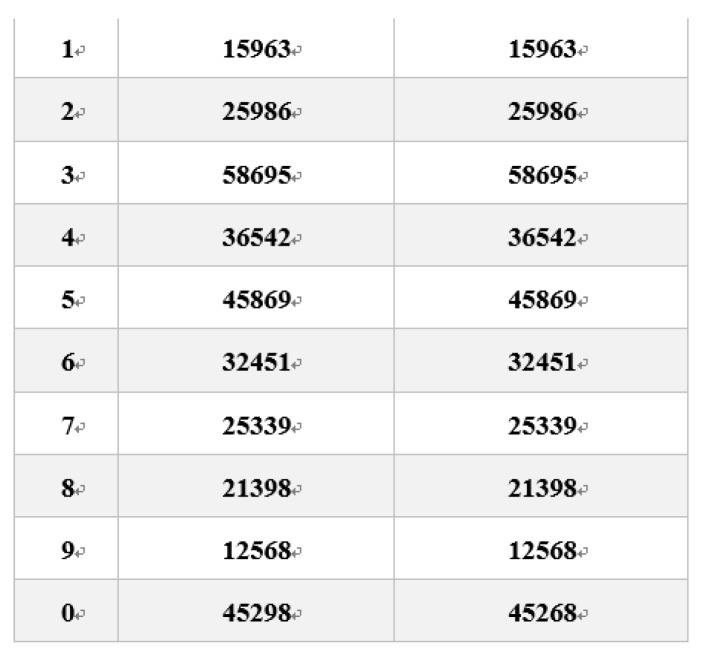
Schematic diagram of the numeral inspection task (NIT).

For the NIT, the steps were as follows [23]: Ten lines of numerals appeared on the computer screen. And each line contained two 5 digits numerals. The subjects were asked to find the only line with different numerals and press the button as soon as possible. The response time (the time interval from the appearance of the numerals to the action of the subject) and the accuracy rate were automatically recorded by the program file. The random numerals of the NIT are shown in Figure 1. Each test consisted of fifteen random tasks.

**Figure 2:**
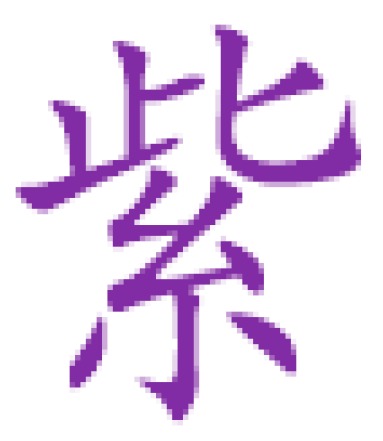
Schematic diagram of the correct result of the SCWT test.

**Figure 3:**
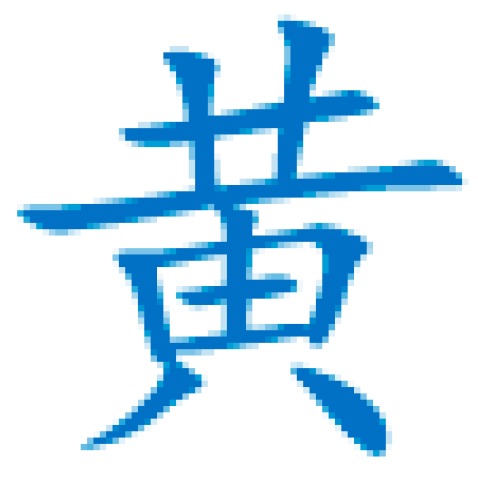
Schematic diagram of the false result of the SCWT test.

For the SCWT, a random colored word appeared at the center of the computer screen, such as black “red”, green “green”, etc. The subjects were asked to judge whether the color of the word was the same as its meaning, then they pressed the button “T” or “F”. “T” suggested that the color of the word was consistent with its meaning, for example, when the word ‘purple’ was shown in purple color, it was “T”. On the contrary, “F” suggested that they were not consistent, such as the blue-color word “yellow”. The response time and the accuracy rate were automatically recorded by the program file. The random words are shown in Figure 2 and Figure 3, and each test consisted of fifteen random tasks.

The NIT and SCWT were conducted by the E-prime program [28].

For grip strength, at each test, the subjects’ instantaneous grip value was measured for two times by an electronic grip meter, then the average value was taken as the test result.

For the evaluation of the work willingness, the thermal comfort and the thermal sensation, a questionnaire survey was conducted. The subjects rated their work willingness by a 7-point scale, where “+3” represented the highest willingness, “−3” represented the lowest willingness [28]. Similarly, the levels of thermal comfort and the thermal sensation were divided into a 5-point and 9-point scale, respectively. And “+2” represented very comfortable, “+4” represented very hot. 

The subjects were asked to practice in advance to be familiar with the above four tests. To avoid the learning effect during the experiment, a total of 200 pictures of NIT and 64 pictures of SCWT were selected in the E-Prime program. Meanwhile, the minimum interval between each temperature condition was four days.

#### 2.1.4. Experiment Process

At each experiment day, all subjects were asked to arrive at the house at 8:30. Then they were requested to rest quietly for 30 min to stabilize their physiological parameters [23]. The experiment started at 9:00. The SCWT, the NIT, and the work willingness questionnaire survey were conducted every hour for 24 consecutive hours. The grip strength, thermal comfort, thermal sensation, and physiological parameters (eardrum temperature, rectal temperature, forehead temperature, heart rate, systolic pressure, and diastolic pressure) were also measured every hour for 24 consecutive hours. Meanwhile, the hourly outdoor temperature was accessed from the website of China Weather [25]. The parameters and instruments in this study are shown in Table 2. During the measurement period, in the day time, the subjects were asked to stay in the house quietly or engage in light mental activities (such as reading or studying); in the night time, they had a short sleep.

The nighttime measurements in the present study focused on the circadian rhythms [23]. However, their effects on the physiological parameters can be ignored as: (1) During night time, subjects adequately had a short sleep between two adjacent measurements (each measurement only took about five minutes); (2) the minimum interval between each temperature condition was four days, and the short-term sleep insufficiency had little effect on physiological circadian rhythms; (3) many studies on circadian rhythms involved nighttime measurements [23,29,30].

**Figure 4:**
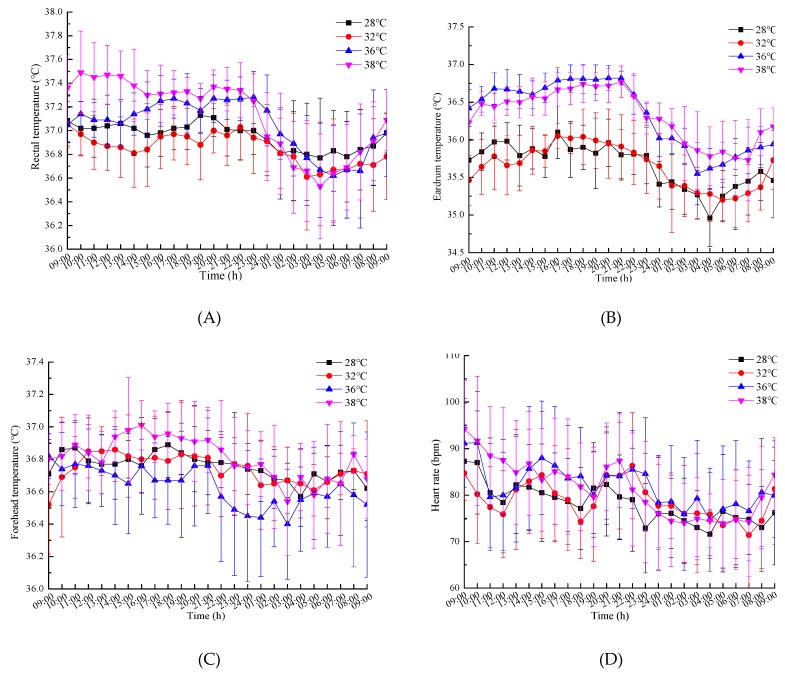

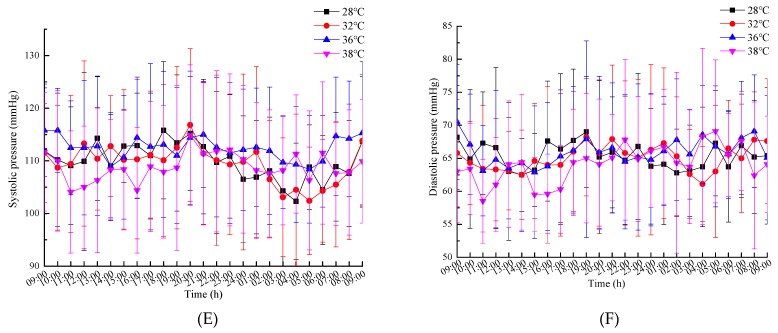
The mean values and standard deviations of the results of the physiological parameters: (**A**) Rectal temperature; (**B**) eardrum temperature; (**C**) forehead temperature; (**D**) heart rate; (**E**) systolic pressure; (**F**) diastolic pressure.

The average values and standard deviations of the results of the physiological parameters are shown in Figure 4.

### 2.2. Body Thermal Balance and Work Efficiency

The thermal balance of the body can be represented as [31]:

(1) M−W−C−R−E−S=0

where M is the energy metabolism rate, W/m^2^; W is the mechanical work done by the human body, W/m^2^; C is the convective heat dissipation between the surface of the human body and the environment, W/m^2^; R is the radiation heat dissipation from the surface of the human body to the environment, W/m^2^; E is the heat taken away by the sweat evaporation and the exhaled water vapor, W/m^2^; S is the heat storage rate, W/m^2^.

In moderate temperature environments, to maintain normal body temperature, the heat production in the body and heat dissipation from the body should be balanced. Thus, the heat storage in the body is zero.

The heat balance of the human body is destroyed due to prolonged exposure to high temperatures. To maintain thermal balance, the heart rate increases to dissipate internal heat to the environment through the skin. Then, the blood vessels widen and skin temperature increases. When the heat cannot be dissipated in time, the heat will be stored in body, and the accumulation of heat leads to an increase of the body temperature, even causing heat induced diseases.

**Figure 5:**
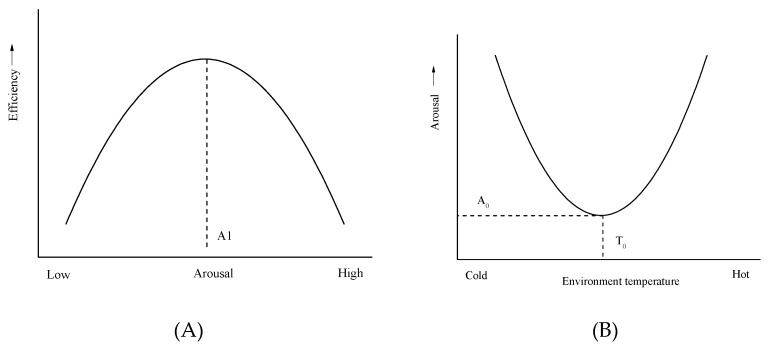
The relationships between: (**A**) Efficiency and arousal; (**B**) arousal and environment temperature.

Arousal is introduced to explain the influence of the environmental stress on work efficiency. Figure 5A gives the relationship between arousal and work efficiency [32]. It indicates that there is an inverted U relationship between arousal and work efficiency. The highest efficiency of work occurs at a moderate level of arousal. At a lower level or a higher level of arousal, the work efficiency drops to a lower level. For environmental stress, cold and heat both cause arousal. A moderate temperature has minimal sensory input to the nervous system, and some studies also indicate that warmth can also reduce arousal, that is, a little warmth often results in a feeling of laziness or weakness. Figure 5B illustrates the relationship between arousal and environment temperature [32]. The minimal arousal temperature corresponds to the temperature of the thermal neutral or slightly higher than the thermal neutral.

In this study, the subjects were in lower labor intensity condition. The metabolic rate reflected the work intensity of human body. In the daytime, they read or rested, and in the night time, they took a short sleep. The work intensities were similar. The energy metabolism rates (M) were similar and relatively low. The heat dissipation through convection (C), radiation (R), and sweat evaporation (E) characterized the human capabilities for thermal adaptation. They were influenced by the environment stress (the arousal). When the ambient temperature exceeds the skin temperature of the human body, there is no convective and radiative heat loss from the body to the environment, and heat dissipation is only performed by sweating. The heat storage rate reflected the body physiological status and human health. In high temperature environments, when heat storage increases, body temperature and sweat increase, and heat induced diseases appear. In addition, the central nervous system is depressed, attention is distracted, and accuracy and coordination decrease, thus work efficiency decreases.

In addition, the subjects were not heat-acclimatized before the experiment. To avoid the formation of heat acclimation, the minimum interval between each temperature condition was 4 days. Therefore, the influence of heat acclimation on work efficiency could be neglected. 

To sum up; based on the relationship between arousal and the environment stress, and using the thermal balance equation, the relationships among human capabilities, human health, environment stress, and work efficiency can be obtained.

### 2.3. Statistical Method

The cosinor analysis is a common method [33] that describes data by a single cosine function with fixed frequency plus a constant (single-harmonic model) yielding amplitude, phase, and mean [34,35]. It is widely used in time medical researches [36,37]. In this paper, the cosinor analysis was adopted to analyze the work efficiency parameters within a 24-hour period. Then, based on the least square principle, the circadian rhythm parameters (P-value, median rhythm, amplitude, acrophase) were measured and analyzed through the Cosinor Periodogram software [38]. According to the results, when P is smaller than 0.05, it suggests that the variable shows circadian rhythms, and when P is larger than 0.05, it indicates that the variable shows no circadian rhythm. The mathematical model of the cosinor analysis is shown in Equation (2) [33]:

(2) $$Y_{i}=M+A\cos(\omega t_{i}+\phi )$$

where $Y_{i}$ is the biological variable at time $t_{i}$; M is the median rhythm (the mean value of rhythm adjustment); A is the rhythmic amplitude (the degree of rhythm variation above or below the central line); ω is the angular frequency of rhythm (considering 24 h circadian cycle as 360°, it is 15°/h); $t_{i}$ represents time of 24 hour system and i can take the values of 0, 1, 2,…, 24; and ϕ is the acrophase of rhythm (the time interval between 0 and the time when the rhythm reaches its peak).

Through SPSS 17.0 software (IBM, Armonk, NY, USA), the paired sample T test was adopted to compare the eigenvalues of the circadian rhythms under these four temperature conditions. When P was smaller than 0.05, it indicated that there was a statistically significant difference. 

## 3. Results

### 3.1. The Effects of Temperature on Circadian Rhythm of Work Efficiency

The single cosinor method of cosinor analysis was used to analyze the rhythms of the subjects’ work efficiency indicators. The cosinor analysis results are shown in Table 3. The results indicated that subjects’ response time in SCWT and NIT, grip strength, and work willingness all had circadian rhythms (*p* < 0.05) under the four temperature conditions, while the accuracy rate in SCWT and NIT did not show significant circadian rhythms (*p* > 0.05).

For the response time in SCWT, the median at 38 °C was significantly larger than those at 28 °C, 32 °C, and 36 °C (*p* < 0.05). In addition, the amplitudes and acrophases among the different temperatures showed no significant difference (*p* > 0.05), and the maximum response time in SCWT appeared between 21:00 and 01:00.

For the response time in NIT, the median at 38 °C was significantly larger than those at 28 °C, 32 °C, and 36 °C (*p* < 0.05). In addition, the amplitudes and acrophases among different temperatures showed no significant difference (*p* > 0.05). The maximum response time in NIT appeared between 21:00 and 03:00, which was similar with that in SCWT.

**Figure 6:**
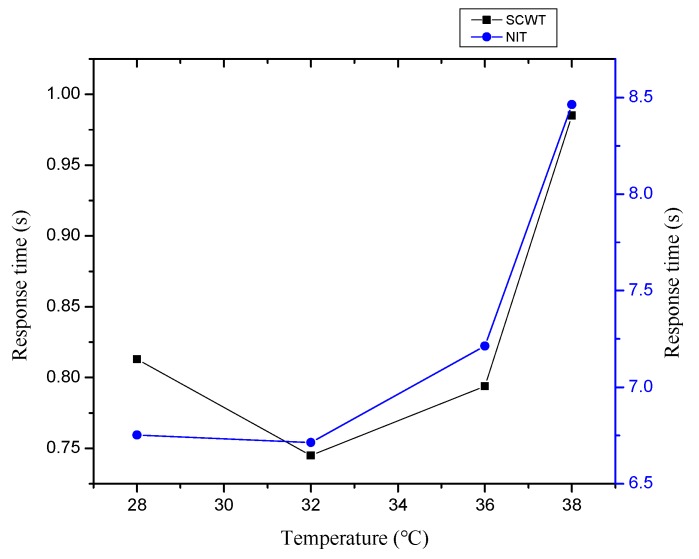
The relationships between the response time and the temperature.

Figure 6 shows the relationships between the response time and the temperature, with the increase of temperature, subjects’ response time showed a change trend of a U-shaped curve.

For grip strength, the medians of subjects decreased with the increase in temperature. The median at 36 °C was significantly smaller than that at 28 °C (*p* < 0.05), while the median at 38 °C was significantly smaller than those at 28 °C and 32 °C (*p* < 0.05). The amplitude at 38 °C was smaller than that at 36 °C. The maximum grip appeared between 15:00 and 18:00.

For the work willingness, the medians decreased with the increase of temperature. And the median of 38 °C was significantly smaller than those at 28 °C and 32 °C (*p* < 0.05). The amplitude at 38 °C was significantly smaller than that at 28 °C (*p* < 0.05). The maximum of the work willingness occurred between 14:00 and 20:00.

As the accuracy rates in the SCWT and NIT did not show significant circadian rhythm, the average values of all subjects at each time point were used to conduct the paired sample T tests to analyze the differences of accuracy rates among different temperature groups. The results of the average accuracy rates are shown in Table 4. The environment temperatures showed a significant effect on the accuracy rates in SCWT and NIT. For the SCWT, there is a significant difference between 28 °C and 32 °C, 28 °C and 36 °C. For the NIT, there is a significant difference between 28 °C and 38 °C, 36 °C and 38 °C.

### 3.2. The Relationships between Work Efficiency and Physiological Indices

**Figure 7:**
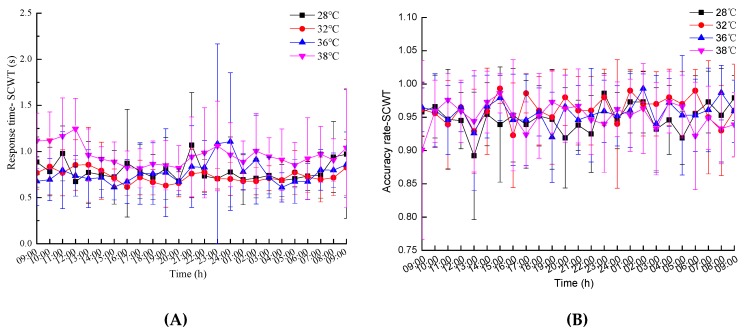

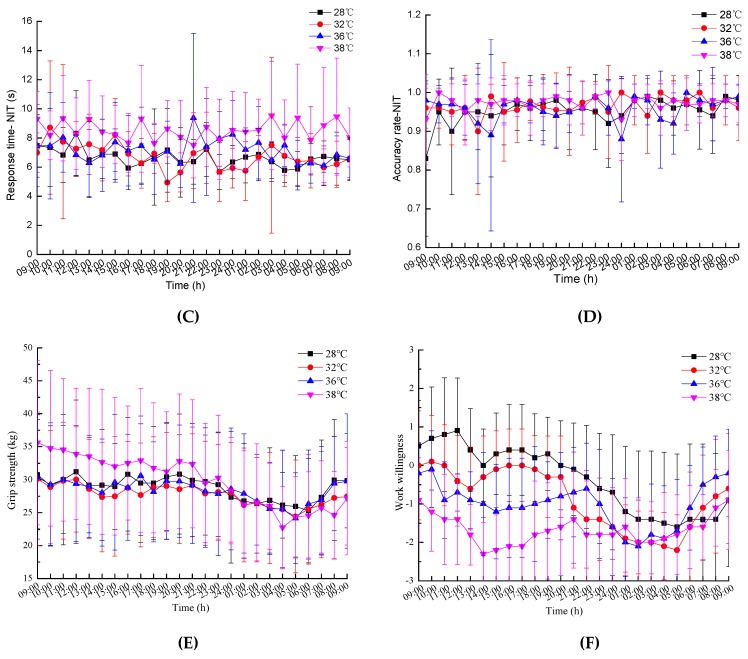
The mean values and standard deviations of the results of the work efficiency indices: (**A**) Response time-SCWT; (**B**) accuracy rate-SCWT; (**C**) response time-NIT; (**D**) accuracy rate-NIT; (**E**) grip strength; (**F**) work willingness.

The average values and standard deviations of the work efficiency indices are shown in Figure 7.

The Spearman rank correlation coefficient was applied to analyze the relationships between the ability indices and the physiological indices. The correlation coefficient was used to estimate the relationship between the two-rank data, and it was frequently used in the analysis of biological experimental data [39] because of its low requirements for data and wide range of applications. Based on Figure 4 and Figure 7, the correlation coefficient results are shown in Table 5. In the SCWT, the response time was negatively correlated to eardrum temperature (in 28 °C and 36 °C conditions) and forehead temperature (in all conditions), while the accuracy rate was not correlated to the physiological indices. In the NIT, the response time was positively correlated to the diastolic pressure (in 28 °C, 32 °C, and 36 °C conditions) and negatively correlated to the eardrum temperature (in in 28 °C, 32 °C, and 36 °C conditions) and forehead temperature (in 32 °C, 36 °C, and 38 °C conditions), and the accuracy rate was correlated to the heart rate (in 28 °C and 32 °C conditions) and the systolic pressure (in 28 °C, 32 °C, and 38 °C conditions). For the grip strength, it was significantly positively correlated to the rectal temperature, diastolic pressure, and systolic pressure in all temperature conditions. For the work willingness, it was significantly correlated to the rectal temperature and eardrum temperature in all temperature conditions.

## 4. Discussion

The efficiency of accomplishing a task is directly related to speed and accuracy. The response time consists of four parts: The time for the sensory organ to feel the stimulus, the time for brain processing, the time for nerve conduction, and the time for the muscle to react [40,41]. As a reliable indicator of mental activity (feeling, attention, learning, memory, thinking, etc.), it reflects the coordination and response capacity of the human nerve and muscle system [42]. The theory of neural activity acceleration [43] argues that the increase of body temperature can accelerate nerve activity and shorten the time it takes for people to perceive the stimulus. Moreover, the effects of environmental stress on wake-up and attention has an inverted U-shaped relationship, and when the level of environmental stress is low, wake-up and attention increase as the temperature rises [44]. In the experiment for SCWT and NIT, the response times at 32 °C were smaller than those at 28 °C ,and the accuracy rates at 32 °C were larger than those at 28 °C (Figure 7). The results are similar with the findings that subjects performed worse during summer conditions of 27 °C than at 32 °C in parts of the tests [39]. 

The application of biological rhythm could help to avoid accidents and working during the peak rhythm was conducive to improve work efficiency. The present study indicated that the circadian rhythms of response time, subjective feelings and physical strength were influenced by high temperature. Under the temperature conditions of 36 °C and 38 °C, the response times in SCWT and NIT significantly increased, and the work willingness and accuracy rates slightly decreased. The reason was that, in indoor high temperature environments, heat stress may cause excessive competition for the total resources of information processing, and then human cognitive behavior is affected [45], then negative emotions increase, the ability of behavior control weakens, the awareness of the reaction to conflicts slows down, and the enthusiasm for work declines. Finally, perception, memory, and recognition changes [46,47,48]. Some studies also indicated that high temperature can significantly reduce the judging interference ability [15,48,49]. In addition, the physiological responses in high temperature environments also made humans feel uncomfortable and weary, and the abilities of perception, memory, and recognition were affected [39,50]. There was a critical temperature (32.2–35 °C), and when temperatures were above this threshold, the performance of mental tasks significantly reduced [51]. 

The local muscle strength and neuromuscular coordination were directly related to the body’s ability to perform manual work. In addition, the circadian rhythms of grip strength were similar with those of the body temperature. For example, the contractile strength of both hands was strongest at 19:00 and weakest at 07:00, while the performance of neuromuscular coordination was the best at 12:00 and the worst at 24:00 [3,52]. The present study also confirmed that the grip strength showed significant circadian rhythms at every temperature condition, and the peak of grip strength appeared between 15:00 and 18:00. In addition, the present study also found that the medians of grip strength significantly decreased at 36 °C and 38 °C, and the amplitude at 38 °C was smaller than that at 36 °C. Therefore, the effects of high temperature on the circadian rhythms of grip strength were mainly reflected in the decreases of the median and amplitude. Tian et al. [14] concluded that although grip fatigue had some impacts on grip strength, the time interval of twenty minutes between two adjacent measurements was sufficient to restore muscle fatigue. In the present study, the time interval was 1 h, therefore the effects of the high temperature on the decline of grip strength could be obtained. High temperature affected grip strength from two aspects. Firstly, body mitochondrial enzyme activity decreased because of the high temperature, as a result, muscle energy supply was insufficient. Secondly, massive sweating caused a large loss of water and electrolytes (mainly calcium ions), and lack of calcium ions led to a decrease of muscle strength, while some symptoms such as limb weakness and cramps appeared [14].

To sum up; the decreases of work efficiency caused by high temperature environments were reflected in prolonged response time, decreased physical strength, and reduced work willingness. The high temperature environments resulted in the decline of the coordination and response capacity of the human body, thus it caused the occurrence of unsafe behavior [53]. For example, the decline in response ability had led to the aggravation of accident proneness of miners [54]. The response time of the accident-prone driver was significantly higher than that of the normal group [55]. In addition, the response speed of the worker was slower under the influence of negative emotions, thus the unsafe production behavior was easily ignored [56]. Therefore, the reduction of work efficiency would threaten the safety of production, and it may cause safety accidents in severe cases. The connection between work efficiency and production safety was conducive to the rational arrangement of work. Wang [57] quantified the relationship between the reduction in work efficiency and work time, and the safety levels corresponding to working efficiency reduction were defined. It was the most dangerous when the work efficiency reduction exceeded 20%, then workers should immediately stop working in the high temperature environments. It was slightly dangerous when the work efficiency reduction was between 10% and 20%, then some warning measures should be immediately taken. And it was safe when the work efficiency reduction was less than 10%, and workers could perform normal work. Based on arm strength, response time, and memory, Kong [50] used the ergonomic deviation rate to further explain the adverse effects of high temperature environments on work efficiency.

The present study found that the eardrum temperature and forehead temperature were both negatively correlated to the response time in SCWT and NIT, and the forehead temperature showed significant negative correlation to the response time even at 36 and 38 °C. The circadian rhythm of forehead temperature usually showed a parallel relationship with the variation of core temperature. Therefore the forehead temperature can be considered as a predictor to assess the level of individual performance. As it is difficult and fussy to measure the ability indices at the work site, the forehead temperature can be considered as an alternative method to replace the direct measurement of the ability indices.

The dynamic monitoring of blood pressure was helpful in the diagnosis of hypertension, and the application of circadian rhythms was more conducive to investigate the information inside the human body. In the present study, the blood pressure of the human body were associated with NIT. Many studies confirmed that the high temperature environment can cause cardiovascular diseases [58,59,60], and the vascular risk factors were negatively correlated to the cognitive function [61]. The high temperature environment affected the body’s cognitive ability from two aspects. Firstly, the high temperature aggravated the occurrence of cardiovascular diseases. Secondly, it caused a decrease in blood pressure. Therefore, it can be concluded that the indoor high temperature affected the cognitive level by causing effects on regulating and setting the circadian rhythm of the body’s blood pressure. This is consistent with the conclusions of the relationships between blood pressure and NIT.

The strengths of the present study lie in the aims and design. Firstly, the study aims to develop a comprehensive method to evaluate the human performance. Response time, subjective feelings, and physical strength were introduced to assess the physical performance from different points of view. Response time, accuracy rate, and grip strength were adopted to evaluate the work efficiency from an objective perspective. And work willingness was adopted to evaluate the work efficiency from a subjective perspective. The application of both subjective evaluation and objective evaluation in the related studies is lacking. Therefore, the application of comprehensive evaluation in the cognitive performance is meaningful. Secondly, the study also aims to reveal the inherent relationships among circadian rhythms, physiological parameters, and ability parameters. It was found that the forehead temperature could be considered a predictor to assess the level of individual performance, and it resolves the disadvantage that the ability indices are difficult to measure at the work site.

The shortcomings of the present study li in: (1) The indoor temperature in this study was dynamic, however the circadian rhythm of the human body also showed stability at a constant ambient temperature, and the influence of the steady environment on the circadian rhythm was not consided in this study, it is worth studying in further research. (2) The subjects in this study were composed of university students. However, the age and the physical quality between the university students and the people in other occupations were different. In further study, subjects from different occupations can be adopted.

## 5. Conclusions

In this paper, the effects of indoor high temperature environments on the circadian rhythms of human work efficiency were studied. In the experiment, the response time, accuracy rate, grip strength, work willingness, and some physiological indicators were measured every hour for 24 consecutive hours. The single cosinor method was used to analyze their basic eigenvalues (median, amplitude, and acrophase) of the circadian rhythms of the ability indicators under the four temperature conditions (28, 32, 36, and 38 °C). The relationships between the ability indicators and the physiological indicators were analyzed by the Spearman correlation analysis method. Conclusions can be listed as follows:

(1) The results of the cosinor analysis indicated that the subjects’ response time, grip strength, and work willingness showed circadian rhythms under these four temperature conditions. However, the accuracy rate did not show a significant circadian rhythm. The paired sample T test was used to analyze the relationships between the accuracy rate and the environment temperature. The results indicated that the environment temperatures had a significant effect on the accuracy rate in SCWT and NIT.

(2) The diastolic pressure was correlated to the response time of the NIT, and the systolic pressure was correlated to the accuracy rate of the NIT. The eardrum temperature and forehead temperature were both significantly negatively correlated to the response time. It was suggested that the forehead temperature could be considered as a predictor to assess the level of individual performance.

(3) The median of the response time in SCWT increased with the increase of temperature, and the relationships between them showed a change trend of a U-shaped curve. In addition, the median at 38 °C was significantly larger than those at 28 °C, 32 °C, and 36 °C. The maximum response time in SCWT occurred between 21:00 and 01:00.

(4) The median of response time in NIT increased with the increase in temperature, and the relationships between them also showed a change trend of a U-shaped curve. More over the median at 38 °C was significantly larger than those at 28 °C, 32 °C, and 36 °C. The maximum response time in NIT appeared between 21:00 and 03:00.

(5) The effects of the high temperature on the circadian rhythms of grip strength reflected in the decrease of the median and amplitude. And the maximum grip strength appeared between 15:00 and 18:00. 

(6) The median of the work willingness decreased with the increase of temperature, and the median at 38 °C was significantly smaller than those at 28 °C and 32 °C. The maximum value occurred between 14:00 and 20:00.

The present study can provide quantitative and basic data for the changes of human work efficiency under indoor high temperature environments. However, the results of this study are limited to young people rather than the elderly as the subjects were college students. In addition, the application of the circadian rhythm is conducive to predict the low-performance of the workers in advance, thereby the incidence of accidents can be reduced.

## Figures and Tables

**Table 1:** Anthropometric information of subjects.

Gender	Age	Height (cm)	Weight (kg)	BMI ^a^ (kg/m^2^)
Male	22.2 ± 1.9 ^b^	172.2 ± 5.1	69.2 ± 12.2	23.2 ± 2.9
Female	23.0 ± 1.4	161.4 ± 4.7	53.8 ± 8.1	20.6 ± 2.9
All	22.6 ± 1.6	166.8 ± 7.3	61.5 ± 12.7	21.9 ± 3.0

Note: (1) ^a^ Body mass index, BMI = weight/height^2^, and the normal value scope is between 18 and 25 kg/m^2^; (2) ^b^ average value ± standard deviation.

**Table 2:** Parameters and instruments.

Parameter	Instrument	Model	Precision
Response time	E-prime software	/	/
Accuracy rate	E-prime software	/	/
Work willingness	Work willingness questionnaire	/	/
Grip strength	Electronic grip meter	CAMRY EH101	±0.5 kg
Heart rate	Electronic sphygmomanometer	OMRON HEM-7051	±5%
Rectal temperature	Electronic thermometer	OMRON MC-347	±0.1 °C
Eardrum temperature	Infrared ear thermometer	OMRON TH839S	±0.2 °C
Forehead temperature	Infrared temperature instrument	DT806	±0.3 °C
Blood pressure	Electronic sphygmomanometer	OMRON HEM-7051	±4 mmHg

**Table 3:** Cosinor analysis results of circadian rhythms of subjects’ work efficiency indices.

Parameters		28 °C	32 °C	36 °C	38 °C
**Response time- SCWT**	M	0.813 ± 0.151	0.745 ± 0.118	0.794 ± 0.201	0.985 ± 0.172 ^▲^^★◆^
	A	0.142 ± 0.071	0.099 ± 0.033	0.204 ± 0.178	0.162 ± 0.055
	ϕ	−190(−121, −259)	−200(−112, −289)	−214(−147, −280)	−237(−150, −323)
	P	0.0291	0.0304	0.0139	0.0320
**Response time- NIT**	M	6.752 ± 1.140	6.714 ± 1.306	7.213 ± 1.085	8.464 ± 1.686 ^▲^^★◆^
	A	1.031 ± 0.393	0.959 ± 0.278	1.292 ± 0.527	1.224 ± 0.615
	ϕ	−218(−158, −279)	−185(−91, −278)	−200(−140, −260)	−263(−222, −305)
	P	0.0197	0.0304	0.0211	0.0158
**Grip strength**	M	29.498 ± 8.154	29.005 ± 8.362	28.612 ± 8.094 ^▲^	28.123 ± 8.139 ^▲^^★^
	A	2.363 ± 0.853	2.237 ± 0.761	3.005 ± 1.632	2.084 ± 1.128 ^◆^
	ϕ	−97(−83, −111)	−122(−78, −166)	−100(−88, −113)	−93(−71, −115)
	P	0.0111	0.0244	<0.0100	0.0110
**Work willingness**	M	−0.741 ± 0.628	−0.837 ± 0.572	−1.108 ± 0.569	−1.743 ± 0.932 ^▲^^★^
	A	1.528 ± 0.837	1.208 ± 0.536	1.095 ± 0.342	0.771 ± 0.337 ^▲^
	ϕ	−115(−46, −183)	−80(−63, −97)	−121(−49, −194)	−157(−74, −241)
	P	<0.0100	<0.0100	<0.0100	<0.0100

Notes: ▲ Indicates that there is a significant difference when the results of 28 °C are compared with those of 32 °C, 36 °C, and 38 °C (*p* < 0.05); ★ indicates that there is a significant difference when the results of 32 °C are compared with those of 36 °C and 38 °C (*p* < 0.05); ◆ indicates that there is a significant difference when the results of 36 °C are compared with those 38 °C (*p* < 0.05); the symbols are the same in the below tables; in this table, the phase of 09:00 is 0 degree; 360° = 24 h.

**Table 4:** Results of the average accuracy rates.

	Accuracy Rate			
	28 °C	32 °C	36 °C	38 °C
**SCWT**	0.94 ± 0.03	0.96 ± 0.20 ^▲^	0.96 ± 0.16 ^▲^	0.95 ± 0.20
**NIT**	0.95 ± 0.03	0.97 ± 0.02	0.96 ± 0.03	0.97 ± 0.02 ^▲◆^

▲ Indicates that there is a significant difference when the results of 28 °C are compared with those of 32 °C, 36 °C, and 38 °C (*p* < 0.05); ◆ indicates that there is a significant difference when the results of 36 °C are compared with those 38 °C (*p* < 0.05).

**Table 5:** Correlation analysis results of work efficiency indices and physiological parameters.

Correlation Coefficient	T *	RT	ET	FT	HR	SP	DP
**Response time - SCWT**	28 °C	0.031	−0.158 ^▼^	−0.157 ^▼^	−0.042	0.228 ^▼▼^	0.170 ^▼▼^
	32 °C	0.041	−0.106	−0.35 ^▼▼^	0.072	0.050	0.046
	36 °C	−0.059	−0.165 ^▼▼^	−0.165 ^▼▼^	−0.013	0.038	−0.106
	38 °C	−0.067	−0.079	−0.151 ^▼^	0.074	0.017	0.043
**Accuracy rate - SCWT**	28 °C	−0.031	0.007	0.039	0.066	0.013	−0.059
	32 °C	−0.110	0.021	0.009	0.064	−0.039	−0.068
	36 °C	0.039	0.086	0.017	0.031	0.045	−0.037
	38 °C	−0.075	−0.012	0.069	0.238	–0.016	0.146
**Response time - NIT**	28 °C	0.049	−0.145 ^▼^	−0.069	0.037	0.119	0.239 ^▼▼^
	32 °C	0.072	−0.187 ^▼▼^	−0.237 ^▼▼^	0.017	0.005	0.167 ^▼▼^
	36 °C	0.131	−0.254 ^▼▼^	−0.433 ^▼▼^	−0.149	0.142	0.271 ^▼▼^
	38 °C	−0.014	−0.085	−0.252 ^▼▼^	−0.088	0.107	0.068
**Accuracy rate – NIT**	28 °C	0.124	−0.015	0.035	0.322 ^▼▼^	−0.280 ^▼▼^	−0.046
	32 °C	0.040	0.100	−0.014	0.205 ^▼▼^	−0.217 ^▼▼^	−0.109
	36 °C	0.007	0.074	0.046	0.052	0.042	−0.004
	38 °C	0.033	−0.088	−0.011	0.267	−0.261 ^▼▼^	−0.149
**Grip strength**	28 °C	0.355 ^▼▼^	0.081	0.243 ^▼▼^	0.052	0.628 ^▼▼^	0.361 ^▼▼^
	32 °C	0.138 ^▼^	−0.025	0.098	−0.255 ^▼▼^	0.677 ^▼▼^	0.428 ^▼▼^
	36 °C	0.352 ^▼▼^	0.291 ^▼▼^	−0.167 ^▼▼^	0.150 ^▼^	0.684 ^▼▼^	0.349 ^▼▼^
	38 °C	0.315 ^▼▼^	0.197 ^▼▼^	−0.031	−0.084	0.617 ^▼▼^	0.182 ^▼▼^
**Work willingness**	28 °C	0.248 ^▼▼^	0.293 ^▼▼^	0.080	0.098	−0.071	−0.016
	32 °C	0.151 ^▼^	0.249 ^▼▼^	0.179 ^▼▼^	0.019	0.177 ^▼▼^	0.211 ^▼▼^
	36 °C	0.356 ^▼▼^	0.142 ^▼^	−0.048	0.151 ^▼^	0.246 ^▼▼^	0.205 ^▼▼^
	38 °C	0.214 ^▼▼^	−0.130 ^▼^	−0.142 ^▼^	0.005	−0.103	−0.023

Notes: (1) ^▼^*p* < 0.05, ^▼▼^*p* < 0.01; (2) * T denotes temperature, RT denotes rectal temperature, ET denotes eardrum temperature, FT denotes forehead temperature, SP denotes systolic pressure, and DP denotes diastolic pressure.
